# Application of structured team model based on shared decision model in obstetrics and gynecology joint intensive care unit (ICU) rescue of critical care pregnant women: A randomized controlled trial

**DOI:** 10.1097/MD.0000000000044430

**Published:** 2025-09-05

**Authors:** Yan Lu, Hua Cai, Liping Chen, Xiaohua Ni, Jianhong Ji, Yaqing Zhou, Yousheng Liu, Yaqiong Jiang, Ying Wang

**Affiliations:** a Department of Obstetrical, Nantong First People’s Hospital, Nantong, Jiangsu Province, China; b Department of Critical Care Medicine, Nantong First People’s Hospital, Nantong, Jiangsu Province, China; c Department of Critical Care Medicine, Hai’an People’s Hospital, Nantong, Jiangsu Province, China.

**Keywords:** critical illness pregnant women, ICU, Obstetrics Department, shared decision-making model, structured team model

## Abstract

**Background:**

This study investigates the clinical value of a structured team approach incorporating shared decision-making in managing critically ill pregnant patients within an obstetrics intensive care unit (ICU).

**Methods:**

A randomized controlled trial was conducted with 100 critically ill pregnant women admitted to our hospital’s obstetrics ICU between January 2023 and December 2024. Participants were allocated via random number table to either the control group receiving conventional multidisciplinary resuscitation care (n = 50) or the observation group receiving the structured team model with shared decision-making (n = 50). Comparative outcomes included resuscitation efficiency indicators (prehospital response time, intrahospital transport duration, and emergency supply preparation time), complication rates, family psychological status measured by Hospital Anxiety and Depression Scale, and family satisfaction assessments.

**Results:**

The observation group demonstrated significantly shorter time metrics across all resuscitation efficiency parameters compared to the control group (*P* < .05). Both groups showed reduced Hospital Anxiety and Depression Scale anxiety and depression scores among family members post-intervention (*P* < .05 vs baseline), with the observation group achieving superior reductions compared to controls (*P* < .05). The structured team model group exhibited significantly lower complication rates (6.00% vs 24.00%, *P* < .05). Family satisfaction assessments revealed higher scores in the intervention group across all domains: medical condition assurance, information accessibility, perceived acceptance, support perception, and comfort maintenance, with total satisfaction scores significantly exceeding those of the control group (*P* < .05).

**Conclusion:**

The implementation of a structured team framework grounded in shared decision-making principles significantly enhances critical care management for obstetric ICU patients. This model improves resuscitation efficiency, reduces complication risks, mitigates family psychological distress, and elevates family satisfaction levels, demonstrating substantial clinical value for high-risk pregnancy care.

## 1. Introduction

The success rate of resuscitation of critically ill pregnant women, that is, those who are at risk of dying during pregnancy, delivery, or within 42 days postpartum, is directly related to the stability of the patient’s family and society.^[1]^ Maternal mortality is also one of the key indicators of global public health concern, and most maternal deaths are avoidable.^[2–4]^ Combined obstetric intensive care unit (ICU) is an important department for resuscitation of critically ill pregnant women and an important safeguard for reducing maternal mortality.^[5,6]^ Traditional multidisciplinary collaboration has problems such as poor communication leading to delayed information, which is prone to affecting the effectiveness of treatment, and the teamwork model should be further optimized. The shared decision-making model is a patient-centered decision-making model that emphasizes the participation of patients, families and medical teams in the decision-making process, and fully considers the opinions and needs of all parties in order to reach the best decision-making solution.^[7,8]^ The application of this model in the medical field has gradually gained attention, which can promote communication and collaboration between medical teams and effectively improve family satisfaction and patient compliance. Compared with the general team model, the structured team model has a clearer team structure, division of responsibilities and workflow, which can improve the team’s collaboration efficiency and decision-making quality.^[9,10]^ The structured team model constructed based on the shared decision-making model is based on the theory of shared decision-making model and integrates the concept of shared decision-making into the structure and operation of the team, so that the team members can participate in the decision-making together under a clear structure and process, give full play to the strengths of various disciplines, and improve the efficiency and quality of treatment. However, the effectiveness of the structured team model based on the shared decision-making model in the rescue of critically ill pregnant women has not yet been fully clarified. Therefore, this study aims to investigate the application of this model in the rescue of critically ill pregnant women in obstetrics combined with ICU, with the aim of providing a reference basis for optimizing the rescue process of critically ill pregnant women and improving the collaborative ability of medical teams.

## 2. Information and methodology

### 2.1. General information

**Figure 1.**
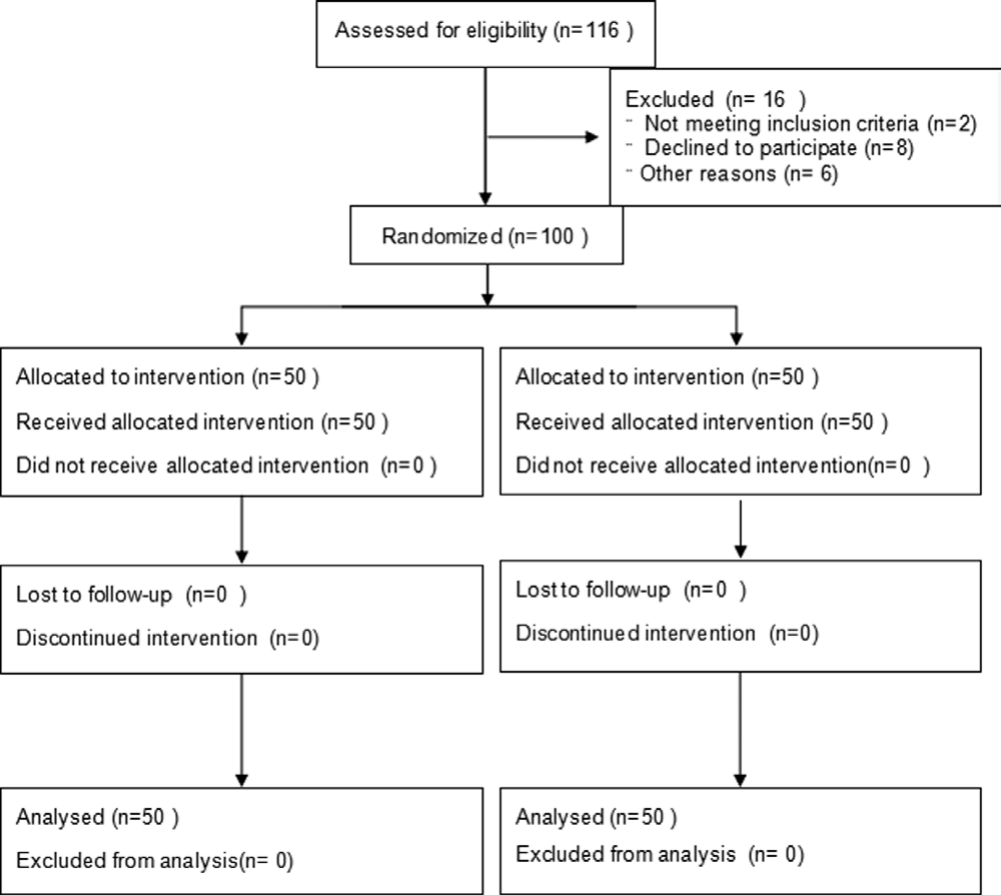
CONSORT flow diagram.

A total of 100 cases of critically ill pregnant women treated in the joint obstetric ICU of our hospital from January 2023 to December 2024 were selected and the patients were divided into the observation and control groups through single-blinded simple randomization using a random number table and the sealed envelope system. Matching was performed for the 2 groups in terms of general characteristics in accordance with the CONSORT guidelines^[11]^ (the guidelines flow diagram is shown in Fig. 1). The sample size is determined using the sample size estimation formula for comparing the mean of 2 samples^[12]^: n1=n2=2*(tα+tβ)*sδ2, with a 0.05 margin of error,and 95% confidence level, α = 0.05, β = 0.1, we can obtained from the *t*-value table: *t*_α_ = 1.96, *t*_β_ = 1.28, and hour *s* = 0.26, δ = 0.17, therefore n=2*(1.96 + 1.28)*0.260.172=49. This study was reviewed and approved by the Ethics Committee of Nantong First People’s Hospital, Ethics Acceptance No. 2022KT096. This study was registered on ClinicalTrials.gov on March 25, 2025, registration number: NCT06930469. Informed consent has been obtained from the participants involved.

Inclusion criteria: Meeting the criteria related to critical care maternity^[13]^: suffering from amniotic fluid embolism, postpartum hemorrhage, or undergoing emergency cesarean section treatment, etc; age ≥ 18 years; gestational weeks > 20 weeks; patients’ families can communicate normally and sign the informed consent.

Exclusion criteria: Combination of primary hematologic diseases; presence of congenital fetal anomalies; combination of malignant tumors; combination of severe hepatic and renal vital function failure; individuals with fuzzy consciousness or mental disorders.

### 2.2. Methodologies

In the control group, routine multidisciplinary rescue care was implemented, and the rescue team was composed of medical and nursing personnel from obstetrics, ICU and related departments, who carried out emergency treatment in accordance with the standard rescue process and division of responsibilities.

The observation group implemented a structured team model based on a shared decision-making model, which operated as follows:

(1) Constructing a structured management team: Multidisciplinary medical and nursing staff, including obstetricians, ICU doctors, obstetric nurses, ICU nurses, head nurses, anesthesiologists, ultrasonographers, and family members of the patient’s main companions, are divided into small teams according to their functions, and each small team has a team leader who is responsible for the coordination of the overall situation and the rapid coordination of information. Obstetricians and ICU doctors are responsible for life support, obstetric evaluation, condition monitoring and development of resuscitation plan for critically ill mothers. Anesthesiologists are responsible for anesthesia management, pain control and intraoperative resuscitation support. The nurse manager coordinates the nursing team to ensure the standardization of rescue care. Obstetrician and ICU nurses were responsible for the execution of specific rescue nursing operations. The patient’s main accompanying family members are responsible for participating in the decision-making process and presenting the wishes and needs of the patient and family members.
(2) Structured team model based on shared decision-making model: (1) Maternal critical care review: Prehospital (prenatal checkup): Obstetricians and nurses conduct regular prenatal checkups for mothers, identify high-risk mothers, and set up high-risk maternal health records. Referral: Critically ill pregnant women establish a green channel for timely referral to the obstetrics department or ICU, and contact the relevant personnel of the structured management team. Assessment: The multidisciplinary team conducts a comprehensive assessment of the extent of the maternal condition, vital signs, and laboratory test results. Identification: Identify the main causes of critical maternal illness and potential risks, such as hemorrhage, infection, and organ failure. Rescue plan: According to the assessment results, formulate a personalized rescue plan and clarify the responsibilities and tasks of each department. Monitoring: Real-time monitoring of maternal vital signs and changes in condition, and timely adjustment of the treatment plan. Records: Detailed records of the rescue process and decision-making basis, to ensure the completeness and traceability of information. (2) Participation of patients’ families in shared decision-making: Information exchange: The management team provides patients and families with detailed information on the condition of critically ill pregnant women and emergency treatment plans, possible risks and prognosis. Understand the patient’s and family’s needs, values and other information, and establish a relationship of trust and respect with the patient. Risk assessment: Assess the risk of the patient’s resuscitation as well as the feasibility and effectiveness of the resuscitation plan according to the specific situation, and provide professional advice. Consideration of values: The patient and his/her family will consider the pros and cons of different resuscitation options according to their own values (e.g., quality of life, economic burden, psychological pressure, etc) and express their own wishes. Selection of options: Based on full understanding and respect for the wishes of the patient, medical personnel and patients and their families to reach the best resuscitation program. (3) Multidisciplinary team participation in shared decision-making to build a structured team: Developing a standardized resuscitation process: Based on clinical practice guidelines, combined with the actual situation of the hospital, develop a standardized process for the resuscitation of critically ill pregnant women. Clearly define the responsibilities and division of tasks of each department and establish a seamless mechanism from prehospital emergency care to in-hospital treatment. The process should cover all aspects of condition assessment, diagnosis, treatment, monitoring, and transfer to ensure the standardization and systematization of rescue work. Team capacity building: Regularly organize multidisciplinary teams to carry out simulation exercises and skills training, and improve the team’s practical ability to rescue critically ill pregnant women through scenario simulation and case analysis. Communication and coordination mechanism: Build a multidisciplinary information sharing platform based on electronic medical record system to realize real-time update and sharing of patient information. We also hold regular multidisciplinary team meetings, where representatives from each department discuss the condition of critically ill pregnant women and the rescue plan, and continuously improve the rescue process and plan.

### 2.3. Observation indicators

Resuscitation efficiency: record prehospital emergency response, in-hospital transfer, and emergency item preparation time.

Family members’ anxiety and depressive mood: assessed using the Hospital Anxiety and Depression Scale (HADS),^[14]^ which has a total of 2 subscales, including anxiety and depression, with 7 entries each, and is rated on a 4-point scale, with a total score of 0 to 21, with higher scores indicating that family members’ anxiety or depressive The higher the score, the more severe the family’s anxiety or depression.

Complications: record complicating events that occurred during resuscitation, such as fever, infection, pelvic hematoma, etc.

Family satisfaction: It was assessed using the Chinese version of the critical care family satisfaction survey,^[15]^ which has a total of 20 entries in 5 dimensions, including assurance of condition, access to information, acceptance, support, and comfort, in the order of 4, 5, 3, 6, and 2 entries, using a 5-point scale with a total score of 20 to 100, with higher scores indicating higher family satisfaction.

### 2.4. Statistical methods

SPSS26.0 software was used to analyze the count data (complications) described by [n (%)] and *χ*^2^ test was used, and the measurement data (resuscitation efficiency, family anxiety and depression, and family satisfaction) were described by (x±s) and *t* test was used, and the difference was considered as statistically significant if (*P* < .05).

## 3. Results

### 3.1. Comparison of baseline data between the 2 groups of robbers

There was no difference in the baseline information of critically ill pregnant women and main accompanying family members between the 2 groups when compared between the groups (*P* > .05), see Table 1.

**Table 1:** Comparison of baseline data between the 2 groups [n (%)] ($\bar{x}\pm \;s$).

Baseline information	Observation group (n = 50)	Control group (n = 50)	*t/χ* ^2^	*P*
Patient				
Age (yr)	30.24 ± 2.37	30.59 ± 2.26	0.756	.452
Week of pregnancy (wk)	34.78 ± 1.68	34.95 ± 1.72	0.500	.618
Number of pregnancies (times)	1.65 ± 0.32	1.53 ± 0.40	1.656	.101
Etiology				
Postpartum hemorrhage	29	31	0.696	.874
Severe preeclampsia	10	9		
Diabetic ketoacidosis	6	7		
Combined heart disease in pregnancy	5	3		
Main accompanying family member				
Character				
Mate	34	37	0.437	.509
Father or mother	16	13		
Age (yr)	37.25 ± 4.56	38.47 ± 3.71	1.467	.145
Education attainment				
Primary and below	7	8	0.361	.835
Middle school	15	17		
College and above	28	25		

### 3.2. Comparison of resuscitation efficiency between the 2 groups

The observation group’s prehospital emergency response, in-hospital transfer, and emergency item preparation time were shorter than that of the control group (*P* < .05), as shown in Table 2.

**Table 2:** Comparison of resuscitation efficiency between the 2 groups ($\bar{x}\pm \;s$, min).

Groups	Number of examples	Prehospital emergency response	Intrahospital transfers	Preparation of first aid items
Observation group	50	6.64 ± 1.05	9.65 ± 2.06	15.14 ± 2.16
Control subjects	50	8.37 ± 1.28	11.27 ± 2.34	18.45 ± 3.27
*t*	–	7.389	3.674	5.972
*P*	–	<.001	<.001	<.001

### 3.3. Comparison of family members’ anxiety and depression in 2 groups

The HADS anxiety and depression subscales of the family members of the patients in both groups were reduced after the intervention compared with the pre-intervention period, and the scores of the HADS anxiety and depression subscales of the family members of the observation group were lower than those of the control group (*P* < .05), as shown in Table 3.

**Table 3:** Comparison of anxiety and depression among family members in the 2 groups ($\bar{x}\pm \;s$, score).

Groups	Number of examples	Anxiety subscale		Depression subscale	
		Pre-intervention	Post-intervention	Pre-intervention	Post-intervention
Observation group	50	15.43 ± 2.18	10.26 ± 1.34*	16.37 ± 1.65	8.95 ± 1.02*
Control subjects	50	15.76 ± 2.04	12.37 ± 2.45*	15.84 ± 2.39	11.20 ± 1.37*
*t*	–	0.782	5.343	1.290	9.315
*P*	–	.436	<.001	.200	<.001

* *P* < .05 compared to pre-intervention.

### 3.4. Comparison of complications between the 2 groups

The complication rate in the observation group (6.00% vs 24.00%) was lower than that in the control group (*P* < .05), see Table 4.

**Table 4:** Comparison of complications between the 2 groups [n (%)].

Groups	Number of examples	Unable think calmly	Infections	Pelvic hematoma	Rate of occurrence
Observation group	50	1 (2.00)	1 (2.00)	1 (2.00)	3 (6.00)
Control subjects	50	5 (10.00)	3 (6.00)	4 (8.00)	12 (24.00)
*χ* ^2^	–	–	–	–	5.020
*P*	–	–	–	–	.025

### 3.5. Comparison of family satisfaction between the 2 groups

All satisfaction ratings (assurance of condition, access to information, acceptance, support, and comfort) and total scores of family members in the observation group were higher than those of the control group (*P* < .05), as shown in Table 5.

**Table 5:** Comparison of family satisfaction between the 2 groups ($\bar{x}\pm \;s$, score).

Groups	Number of examples	Sickness guarantee	Access to information	Acceptance	Favour	Comforts	Total
Observation group	50	17.24 ± 1.32	22.16 ± 1.45	13.89 ± 0.98	27.45 ± 1.63	8.76 ± 0.72	89.50 ± 3.15
Control subjects	50	14.56 ± 1.17	19.87 ± 1.23	11.23 ± 0.89	23.54 ± 1.56	6.54 ± 0.67	75.74 ± 2.98
*t*	–	10.744	8.516	14.208	12.254	15.961	22.438
*P*	–	<.001	<.001	<.001	<.001	<.001	<.001

## 4. Discussion

Common acute and critical perinatal conditions include postpartum hemorrhage, preeclampsia, diabetic ketoacidosis, etc, and in severe cases, shock or multi-organ failure may even occur, which may then endanger maternal life.^[16]^ Timely and effective resuscitation treatment is essential to save maternal life. The treatment of critically ill pregnant women involves obstetrics, ICU and other departments, and the delay in information transfer between departments due to untimely information transfer, single communication method and professional differences can easily lead to the delay in information transfer, which in turn affects the timeliness of treatment decisions.^[17,18]^ And it is equally crucial to improve the collaborative ability of multidisciplinary rapid response teams in the treatment of critically ill pregnant women.

The shared decision-making model emphasizes the participation of patients and their families as well as multidisciplinary teamwork. In the treatment process, patients and their families need to fully understand the condition and treatment plan, and express the wishes and needs of the affected party, which is conducive to the clinical development and selection of a treatment plan that is more in line with the actual situation of the patient.^[19–21]^ The collaboration of multidisciplinary team, that is, by integrating the professional resources of each department, the medical and nursing staff of each department jointly participate in the decision-making process to ensure the scientificity and rationality of the treatment plan.^[22,23]^ And the structured team model based on the shared decision-making model. By constructing a structured management team, the duties of the members are clearly defined; standardized resuscitation procedures are formulated to regulate the resuscitation work; simulation exercises and skills training are regularly carried out to improve the team’s ability; and real-time updating and sharing of patients’ information is achieved through informatization means such as the electronic medical record system, which improves the accuracy of decision-making.^[24]^ This study showed that compared with the control group, the observation group had a shorter prehospital emergency response, in-hospital transfer, and emergency item preparation time, and a lower complication rate. Han Qiong et al^[25]^ reported that the implementation of emergency care under the guidance of shared decision-making model can improve the efficiency of emergency care for patients. The reasons considered were that the construction of a multilevel team under the structured team model based on the shared decision-making model further clarifies the responsibilities and task division of each member, enables each member to perform their own duties and work together, flexibly allocates resources according to the patient’s specific situation and changes in the condition, and reduces the waste of time due to unclear responsibilities or delayed decision-making, thus improving the efficiency and quality of resuscitation; at the same time, through the development of simulation exercises and At the same time, through simulation drills and skills training, the team’s rescue experience and collaboration ability are improved, accurate and rapid judgment is made, and the risk of complications during treatment is reduced as much as possible. This study shows that compared with the control group, the families of the observation group had lower scores on the HADS anxiety and depression subscales. Lucy Deng et al^[26]^ reported that shared decision-making improved patients’ psychological functioning when implemented. The reason considered was that because of the critical condition of the pregnant woman and the relatively depressing environment, it is easier to aggravate the family’s worry, anxiety and depression about the patient’s condition. The clear division of labor in the structured team model based on the shared decision-making model can enhance the team’s ability to work together, so that the rescue work can be carried out in an orderly manner, and strengthen the family’s confidence in the rescue work; at the same time, the patient’s main family members, as one of the team members, can participate in the decision-making process, communicate with the team, and put forward the wishes and needs of the family members of the patient, so as to reduce the anxiety and depression caused by helplessness and uncertainty. This study showed that compared to the control group, the family members of the observation group had higher satisfaction ratings (assurance of condition, access to information, acceptance, support, and comfort) and total scores in each category. The reason for this was considered to be the shared decision-making model in which the healthcare provider and the patient make decisions together, and the patient weighs the risks and disadvantages of treatment options based on full access to information and makes the best decision based on values and preferences. This process fully respects the wishes of the patient, which can make the family feel respected, thus increasing the sense of identity and satisfaction with the treatment process; at the same time, the structured team can promote communication and collaboration among team members, so that the family feels the cohesion and professionalism of the team, which can further improve the satisfaction evaluation.

In conclusion, the structured team model based on the shared decision-making model applied to the obstetrics combined ICU in the rescue of critically ill pregnant women has a significant effect, which can effectively improve the efficiency of the rescue, prevent complications, improve the patient’s family anxiety and depression, and improve the family satisfaction evaluation.

## Author contributions

**Conceptualization:** Yan Lu, Hua Cai, Yaqing Zhou, Yaqiong Jiang, Ying Wang.

**Data curation:** Yan Lu.

**Formal analysis:** Jianhong Ji, Ying Wang.

**Funding acquisition:** Yan Lu, Yousheng Liu.

**Investigation:** Hua Cai, Liping Chen.

**Methodology:** Hua Cai, Liping Chen, Ying Wang.

**Project administration:** Xiaohua Ni, Ying Wang.

**Resources:** Liping Chen, Xiaohua Ni, Jianhong Ji, Yaqing Zhou, Yousheng Liu.

**Software:** Yaqing Zhou, Ying Wang.

**Supervision:** Liping Chen, Yousheng Liu.

**Validation:** Liping Chen, Xiaohua Ni.

**Writing – original draft:** Yan Lu, Hua Cai, Yaqiong Jiang, Ying Wang.

**Writing – review & editing:** Yaqing Zhou, Yaqiong Jiang, Ying Wang.
